# Water, in Disease, &c.

**Published:** 1855-03

**Authors:** J. C. Rutherford

**Affiliations:** Blackstone, Mass.


### WATER, AS A THERAPEUTIC AGENT AND A CAUSE OF DISEASE.

BY J. C. RUTHERFORD, M. D., BLACKSTONE, MASS.

Water, in its purity, is one of the greatest blessings bestowed upon mankind; it is a part of ourselves, and enters largely into all animal and vegetable substance. We must have it for most of the purposes of life. It enters in a large proportion, into the composition of the blood. Whatever it may hold in solution, must become a portion of that fluid, either for good or evil.

It is a fact that can be demonstrated, that water contains more of the elements of disease, than the air we breathe or the food we eat. This applies more particularly to the hot season. Any one who has examined the contents of a cistern or well, is aware of the strong stench of the water and dirt at the bottom. This stench is owing to the animal matters that settle at the bottom, and there form a mass of putrid carrion.

It is not necessary for me to state that rain, river and sea water contain an immense number of animalculi, that cannot be seen without the aid of the microscope. These are short lived, and from their great numbers, form one fourth of the deposit in our wells, cisterns and beds of rivers.

In warm weather this mass undergoes decomposition, and hence the water is an essence of putrefaction. The less water there is in our wells, cisterns, &c. the more concentrated becomes this essence. If any one doubts this, let him go to any well or cistern, or, if he pleases, to the river, and he will find, particularly in the hot season, this fœtid smell, even if the water is not agitated.

To say that such water is not injurious to the system, or that it is not a cause of disease, would be as absurd as to say that putrid flesh and rotten vegetables are wholesome food. Of all the causes that are supposed to produce the cholera, dysentery, diarrhœa, cholera morbus, &c., this may be regarded as the chief. These diseases are most prevalent in hot and dry weather, and they are more or less virulent as the drought is or is not severe. Water we must have, as an element in the animal economy, and as a therapeutic agent; but as we often find it at this season, it is neither one nor the other.

It is allowed on all hands that the effluvia arising from decaying animal and vegetable matter is a great cause of cholera, and other diseases of the bowels. Now if this is a fact, how much more detrimental to health would be the taking into the system this poison in substance; or, in other words, drinking water holding more or less of this matter in solution? Yet we do this when we drink water from our wells and cisterns in the hot summer months. No constitution can endure this contamination a great while. Districts that are most favorable to the existence of intermittent fever, will be found to be those where the cholera shows its most virulent character. The localities most likely to produce these poisons, are low, moist, and fertile, with but little chance for drainage, either by art or nature. The moist and warm soil is favorable for the growth of vegetable matter and animalculi—generation after generation of which are produced and reproduced in one hot season. They have brief existence, and die, forming a mass of putrid matter, to be dissolved in the pools, springs and streams; and that which remains upon the soil, to be dissolved by the dews of night, and as vapor to be wafted about by currents of air.

People who live in malarious districts, are cognizant of the fact that a hard frost puts an end to all danger from miasm for the season. Cold will destroy the poison of the most contagious disorders; and it must be that the frost destroys the effluvia from decomposing matters, and at the same time puts a stop to further decomposition. It is known that cold will put a decided check upon the cholera.

Now the natural inference to be drawn from these facts would be, that all the disorders that have been mentioned are caused by an animal poison that is dissolved in the water we drink, and in the damp air of the night. Another fact tending to establish this theory is, that nine-tenths of the attacks of cholera, dysentery, and other bowel complaints, occurr at night. Dampness seems to be an essential element for the rapid absorption of the poison, and hence the frequency of the attack in the night.

It has been said that water holding lime in solution is a great cause of bowel complaints; but this is not established. There are localities where the water is highly impregnated with lime, but where dysentery is decidedly rare, and the cholera never known. There are other localities where there is hardly a trace of lime, and where diseases of the bowels are the most prevalent disorder. Water which holds the most lime in solution, contains the least animal matter.

The rapid decomposition of animal matter in water, can be demonstrated by taking a vessel of the purest well or cistern water that can be obtained, and setting it where the sun can have full play upon it for a few hours, when you will have the same putrid smell and taste—but in a less degree—that is found in the bottom of our wells. Set water from the same fountain, in a place where the temperature is unfavorable for decomposition, and you will have no perceptible change in taste or smell.

How shall we make this element, that is so often a cause of disease, a harmless and useful article? As one means, it is the duty of physicians to enjoin upon the people the necessity of having every well, cistern, or spring, containing water used for domestic purposes, thoroughly cleansed at least once a year. This will make the water comparatively good. But for a beverage, and especially for invalids, water should be boiled, as a great share of the impurities will then pass over in the vapor, and the more solid parts meet with a chemical change, which renders them inert. Thus it becomes a pleasant and harmless drink, and powerful auxiliary in the treatment of disease. This is a matter that calls for our most careful investigation and serious consideration.—*Boston Medical and Surgical Jour.*

